# The Responsiveness of TrkB to BDNF and Antidepressant Drugs Is Differentially Regulated during Mouse Development

**DOI:** 10.1371/journal.pone.0032869

**Published:** 2012-03-02

**Authors:** Antonio Di Lieto, Tomi Rantamäki, Liisa Vesa, Sudhirkumar Yanpallewar, Hanna Antila, Jesse Lindholm, Maribel Rios, Lino Tessarollo, Eero Castrén

**Affiliations:** 1 Sigrid Jusélius Laboratory, Neuroscience Center, University of Helsinki, Helsinki, Finland; 2 Neural Development Section, Mouse Cancer Genetics Program, Center for Cancer Research, NCI, Frederick, Maryland, United States of America; 3 Department of Neuroscience, Tufts University School of Medicine, Boston, Massachusetts, United States of America; Nathan Kline Institute and New York University School of Medicine, United States of America

## Abstract

**Background:**

Previous studies suggest that the responsiveness of TrkB receptor to BDNF is developmentally regulated in rats. Antidepressant drugs (AD) have been shown to increase TrkB signalling in the adult rodent brain, and recent findings implicate a BDNF-independent mechanism behind this phenomenon. When administered during early postnatal life, ADs produce long-lasting biochemical and behavioural alterations that are observed in adult animals.

**Methodology:**

We have here examined the responsiveness of brain TrkB receptors to BDNF and ADs during early postnatal life of mouse, measured as autophosphorylation of TrkB (pTrkB).

**Principal Findings:**

We found that ADs fail to induce TrkB signalling before postnatal day 12 (P12) after which an adult response of TrkB to ADs was observed. Interestingly, there was a temporally inverse correlation between the appearance of the responsiveness of TrkB to systemic ADs and the marked developmental reduction of BDNF-induced TrkB in brain microslices *ex vivo*. Basal p-TrkB status in the brain of BDNF deficient mice was significantly reduced only during early postnatal period. Enhancing cAMP (cyclic adenosine monophosphate) signalling failed to facilitate TrkB responsiveness to BDNF. Reduced responsiveness of TrkB to BDNF was not produced by the developmental increase in the expression of dominant-negative truncated TrkB.T1 because this reduction was similarly observed in the brain microslices of *trkB.T1* ^−/−^ mice. Moreover, postnatal AD administration produced long-lasting behavioural alterations observable in adult mice, but the responses were different when mice were treated during the time when ADs did not (P4-9) or did (P16-21) activate TrkB.

**Conclusions:**

We have found that ADs induce the activation of TrkB only in mice older than 2 weeks and that responsiveness of brain microslices to BDNF is reduced during the same time period. Exposure to ADs before and after the age when ADs activate TrkB produces differential long-term behavioural responses in adult mice.

## Introduction

Brain-derived neurotrophic factor (BDNF) and its receptor TrkB are critical regulators of neuronal survival, differentiation, axonal and dendritic growth, and synapse formation [1]. Within the last years, their role in the adult central nervous system (CNS), regulating functions such as neuronal plasticity, cognition, anxiety and mood regulation, has been elucidated [2]–[5].

BDNF and TrkB play a central role in the mechanism of action of antidepressant drugs (AD) [6], [7]. All clinically used ADs rapidly induce the autophosphorylation of TrkB and this TrkB activation seems to be a common step in the mechanism of action of all ADs [8], [9]. Behavioural effects of ADs are blunted in animals with reduced levels of BDNF or TrkB signalling in the CNS [8]. On the other hand, infusion of BDNF into the adult brain or over-expression of TrkB in neurons produces an AD-like behaviour in rodents [10]–[12]. These data suggest that BDNF-TrkB signalling is both necessary and sufficient for the AD-induced behavioural effects in adult rodents.

Previous finding suggests that the responsiveness of TrkB to BDNF is regulated during development; BDNF readily activates TrkB in early postnatal rat brain microslices, but this effect of BDNF is strongly blunted after the second week postnatally [13]. This finding is in contrast with the fact that BDNF levels in brain increase rapidly during this period [14], which corresponds to the end of the period of the most intense neuronal migration and differentiation.

Exposure to ADs during early life has been shown to produce long-lasting behavioural changes in rodents that are evident even in adulthood. Specifically, depressive and anxiety-like, motor and sexual behaviours are modified in adulthood [15]–[17] and these changes can be ameliorated by adult AD treatment [17]. AD treatment during postnatal days 4–21 produces long-term changes in *bdnf* and *trkB* mRNA expression in mice [17], which suggest that BDNF-TrkB signalling may play a role in the long-term behavioural consequences of AD treatment in early life.

We have here studied the phosphorylation response of TrkB to systemic ADs and *ex vivo* BDNF at different stages of mouse postnatal development. Our results suggest the intriguing hypothesis that during the postnatal development, there is a shift in TrkB responsiveness from a receptor that is readily activated by BDNF but refractory to ADs to a TrkB clearly phosphorylated by ADs but only weakly activated by BDNF. This shift may play a role in the development of certain adult emotional phenotypes.

## Results

### Systemic imipramine treatment activates brain TrkB signalling in an age-dependent manner

We have previously shown that a single intraperitoneal (i.p.) injection of AD induces phosphorylation of TrkB within an hour in the prefrontal cortex (PFC) and hippocampus (HC) of adult rodent brain [8], [9], [18]. In this study, we treated mouse pups aged between P5 and P21 with a single i.p. injection of either saline or antidepressant imipramine (IMI; 30 mg/kg) and analyzed the phosphorylation status of distinct tyrosine residues of TrkB at 30 min after the injection.

**Figure 1:**
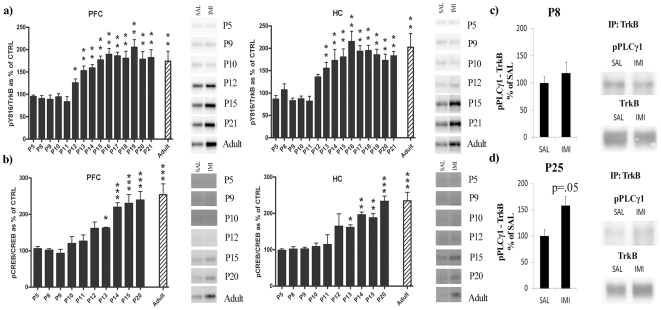
Age-dependent effect of systemic imipramine on TrkB phosphorylation and signaling in the mouse brain. (a) Phosphorylation of TrkB phospholipase-Cγ1 (PLCγ1) binding site (Y816) after acute imipramine treatment (30 mg/kg, 30 min, i.p.) in prefrontal cortex (PFC) and hippocampus (HC). Phospho-TrkB values are normalized against total TrkB levels. (b) Phosphorylation of CREB (Ser^133^) after acute imipramine treatment (30 mg/kg, 30 min, i.p.) in prefrontal cortex (PFC) and hippocampus (HC). Phospho-CREB values are normalized against total CREB levels. (c) The effect of acute imipramine treatment (30 mg/kg, 30 min, i.p.) on the association of phosphorylated PLCγ1 (Tyr^783^) with catalytic TrkB receptors in P8 mouse pup hippocampus. (d) The effect of acute imipramine treatment (30 mg/kg, 30 min, i.p.) on the association of phosphorylated PLCγ1 (Tyr^783^) with catalytic TrkB receptors in P25 mouse pup hippocampus. Results are expressed as percentage of respective control. A t-test was performed between each control and treated group of animals at the different ages; *P<0.05, ** *P*<0.01, *** *P*<0.001. n = 6–7 per group.

Our results showed that, along postnatal period, TrkB response to ADs is age-dependently regulated. In the PFC, acute IMI treatment of mice between P5 and P11 failed to increase TrkB phosphorylation at the PLCγ1 site (pY816) (**Figure 1a**). However, a statistically significant increase of pY816-band immunoreactivity was observed from P12 until adulthood in IMI treated animals when compared to their saline treated controls (**Figure 1a**). In the HC, TrkB response to acute IMI treatment presented a similar activation pattern (**Figure 1a**). In both tissues, the magnitude of phosphorylation of the Y816 during the late stage of postnatal period was similar to the one observed in adult animals.

We have previously shown that ADs specifically induce phosphorylation of TrkB at the PLCγ1 site (Y816) and the phosphorylation of CREB (cAMP response element binding protein), the downstream target of this pathway, whereas no increase in phosphorylation levels is observed at the Shc site (pY515) or its downstream target Akt [9]. Consistent with this, acute IMI treatment induced a significant increase in brain pCREB levels in mice only after P13 (**Figure 1b**). This temporal pattern of CREB activation closely followed that of TrkB phosphorylation. IMI treatment also induced the association of phosphorylated PLCγ1 with catalytic TrkB receptors in P25 mouse pups whereas no such association was observed at P8 (**Figure 1c–d**). In contrast, IMI administration did not influence the phosphorylation of Y515 site or Akt in PFC or in HC at any of the age points investigated during the postnatal period (**Figure S1**). Furthermore, as already reported in adult brain [9], [18], acute treatment with IMI did not regulate total protein levels of TrkB at any age (**data not shown**).

### Age-dependent BDNF-TrkB signalling in mouse brain samples

**Figure 2:**
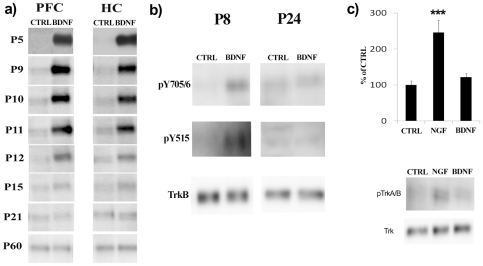
Developmental regulation of BDNF-induced TrkB receptor phosphorylation. (a) Representative blots of experiments showing age-dependent modification of BDNF-induced (50 ng/ml, 5 min, at 37°C) TrkB tyrosine phosphorylation (Y816) response in mouse cortical (PFC) and hippocampal (HC) microslices *ex vivo*. (b) Representative blots of experiments showing that BDNF (50 ng/ml, 15 min, 37°C) readily induces TrkB phosphorylation at sites Y515 and Y705/6 in P8 hippocampal microslices whereas neither site is effectively phosphorylated by BDNF in P24 hippocampal microslices. (c) NGF (50 ng/ml, 15 min, at 37°C) readily induces TrkA tyrosine phosphorylation (Y674/5) in P24 hippocampal microslices whereas BDNF (50 ng/ml, 15 min, at 37°C) has no effect on TrkB phosphorylation (Y705/6) under the same conditions. For statistical analysis, a one-way ANOVA followed with Bonferroni *post hoc* test was performed; ***P<0.001. n = 4 per group. Phospho-TrkB values are normalized against total TrkB levels.

Knüsel *et al* [13] reported that incubation of hippocampal and cortical microslices prepared from rat pups up to the age of P7 respond to BDNF exposure with a robust increase in TrkB phosphorylation. However, starting from P14 and in the adult brain, this response of TrkB to BDNF is significantly reduced [13]. Consistent with these observations, incubation of fresh PFC and HC microslices with BDNF elicited a strong increase of pTrkB levels at P5 (**Figure 2a**). This response is still present at P9 and P11 (**Figure 2a**). However, a clear decline in BDNF-induced pTrkB levels was observed between P11 and P12 and only a modest increase in pTrkB levels was detectable at P15 or after (**Figure 2a**). The ability of BDNF to elicit phosphorylation of TrkB in brain microslices prepared from adult mice (P60) was completely abolished (**Figure 2a**), consistent with previous findings [13]. After an acute treatment with BDNF no differences were found in total protein levels of TrkB at any age (**Figure S2**).

The studies by Knüsel *et al* [13] assayed the overall tyrosine phosphorylation of TrkB after BDNF application, whereas we have assayed the phosphorylation status of a specific tyrosine residue in TrkB, the Y816 site. Consequently, we tested whether also the other tyrosine residues in TrkB are hypo-responsive to BDNF in late postnatal period. Indeed, significantly reduced BDNF –induced phosphorylation of Y515 and Y705/6 (catalytic domain) sites of TrkB was seen in hippocampal microslices obtained from P24 mice but at P8 there was a clear induction in both sites (**Figure 2b**). Essentially similar developmental hyporesponsiveness of Y515 and Y705/6 sites of TrkB was seen in hippocampal microslices prepared from rat pups (P7-P27) (**data not shown**).

The developmentally reduced ability of BDNF to induce TrkB phosphorylation in the mouse brain microslices is probably not due to reduced penetrance of BDNF into mature brain tissue since a highly related neurotrophin, NGF (nerve growth factor), readily induced TrkA phosphorylation (Y674/5) in hippocampal microslices obtained from P24 mice (**Figure 2c**).

**Figure 3:**
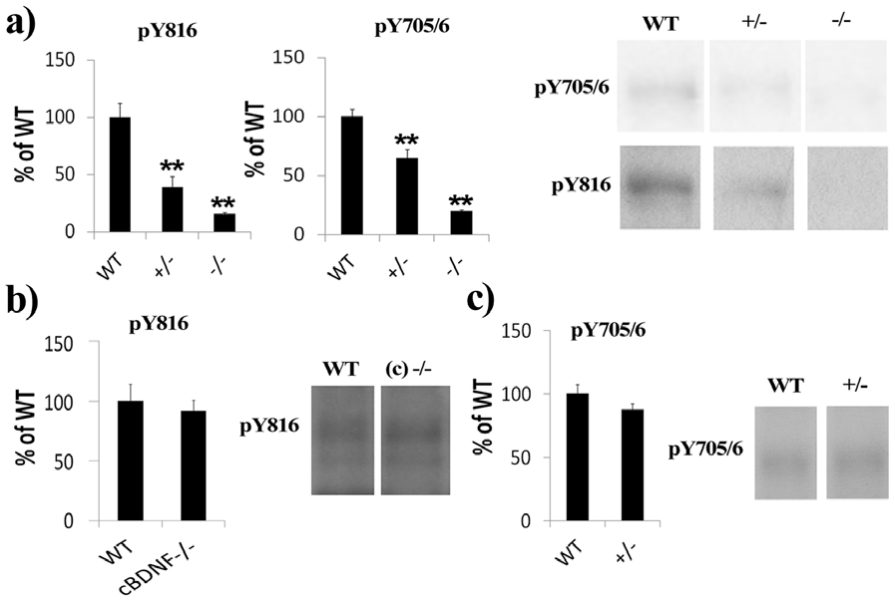
Immature, but not adult, BDNF deficient mice show reduced basal hippocampal TrkB phosphorylation. (a) Basal hippocampal TrkB phosphorylation (Y816, Y705/6) is significantly reduced in P11 old *Bdnf* ^+/−^ (+/−) and *Bdnf* ^−/−^ (−/−) mouse pups compared to age-matched wild-type (WT) mice. (b) Basal hippocampal TrkB phosphorylation (Y816) is not altered in mice with conditional deletion of BDNF in the forebrain (c*Bdnf* ^−/−^ or (c) −/−). (c) Basal hippocampal TrkB phosphorylation (Y705/6) is not altered in adult *Bdnf* ^+/−^ (+/−) mouse. For statistical analysis, a t-test was performed; **P<0.01. n = 3–6 per group. Phospho-TrkB values are normalized against total TrkB levels.

In order to examine the *in vivo* developmental responsiveness of TrkB to BDNF, we analysed basal TrkB phosphorylation status in the hippocampi of immature and adult mice with reduced BDNF levels. In line with the *ex vivo* experiment, basal TrkB phosphorylation at Y816 and Y705/6 sites was significantly reduced at P11 in *Bdnf* ^+/−^ mouse pups [19] when compared to age-matched wild-type (WT) mice (**Figure 3a**). pTrkB levels were almost undetectable in samples obtained from P11 *Bdnf* ^−/−^ mouse pup hippocampi (**Figure 3a**). However, basal TrkB phosphorylation levels were unaltered in adult mice with conditional deletion of forebrain BDNF (c*Bdnf* ^−/−^) [20] (**Figure 3b**). Moreover, and as shown before [21], basal TrkB phosphorylation was unaltered in the hippocampus of adult *Bdnf* ^+/−^ mice (**Figure 3c**).

### Role of TrkB.T1 in regulating the responsiveness of TrkB to BDNF and imipramine

**Figure 4:**
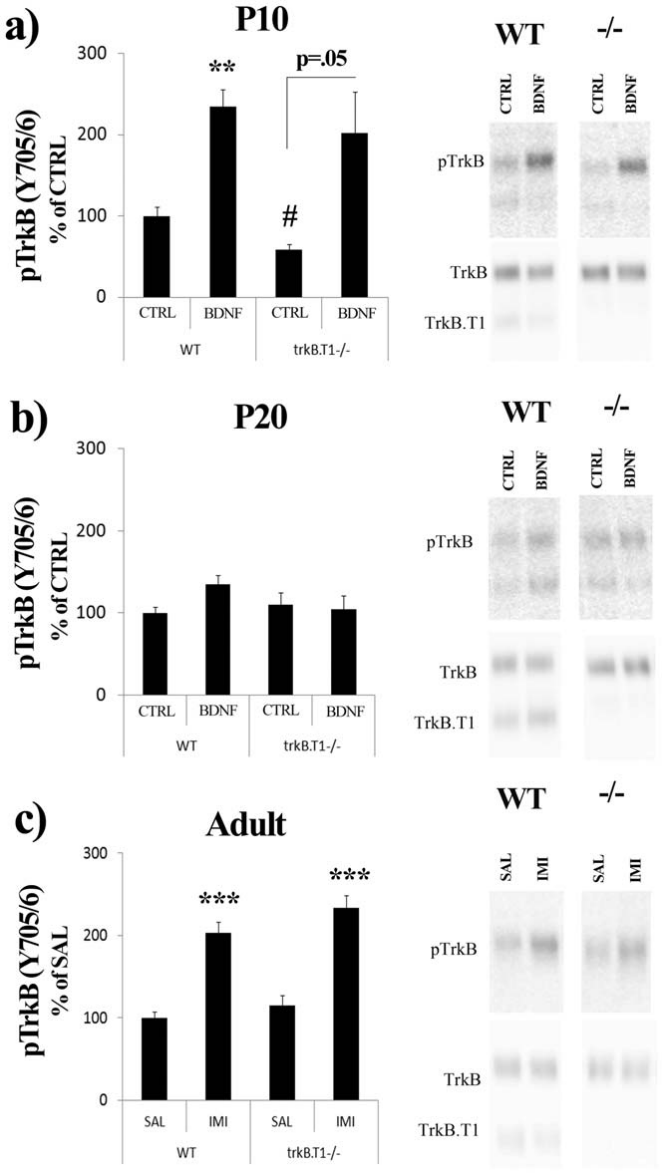
TrkB responsiveness to *ex vivo* BDNF and systemic imipramine is not altered in *trkB.T1* ^−/−^ mice. BDNF-induced (*ex vivo*, 50 ng/ml, 5 min) TrkB phosphorylation (Y816) in hippocampal microslices prepared from P10 (a) or P20 (b) wild-type and *trkB.T1* ^−/−^ KO pups. (c) Imipramine-induced (30 mg/g, i.p., 30 min) TrkB phosphorylation (Y816) in adult male wild-type and *trkB.T1* ^−/−^ mouse hippocampus. Two-Way ANOVA followed with Bonferroni *post hoc* test was performed for statistical analysis; ** *P*<0.01, *** *P*<0.001 compared to respective control, #*P*<0.05 compared to wt/control. The CTRL bar represents control treatments at each age. Phospho-TrkB values are normalized against total TrkB levels. n = 3–5 per group.

Since the developmental increase in the expression of TrkB.T1, the predominant truncated TrkB receptor, coincides with the reduced responsiveness of TrkB receptor to BDNF [13], it has been suggested that TrkB.T1, acting as dominant-negative partner for full-length TrkB and as BDNF scavenging receptor [22], [23], might explain the reduced responsiveness of TrkB to BDNF during late postnatal development [13]. We therefore investigated the responsiveness of TrkB to BDNF and IMI treatments in WT and *trkB.T1* ^−/−^ knock-out mice [24]. Incubation of brain microslices with BDNF produced an age-dependent down-regulation of TrkB phosphorylation levels from P10 to P20 in both *trkB.T1* ^−/−^ knock-out and in WT mice (**Figure 4a–b**). These data suggest that developmental increase in TrkB.T1 expression does not explain the developmental loss of TrkB responsiveness to BDNF. Unexpectedly, basal TrkB phosphorylation level in P10 *trkB.T1* ^−/−^ knock-out hippocampal tissue was significantly reduced compared to WT mice (**Figure 4a**). Acute systemic IMI treatment produced an essentially similar increase in TrkB phosphorylation in adult WT and *trkB.T1* ^−/−^ knock-out mice (**Figure 4c**).

### Potential facilitation of BDNF-TrkB signalling by intracellular cAMP and antidepressants

**Figure 5:**
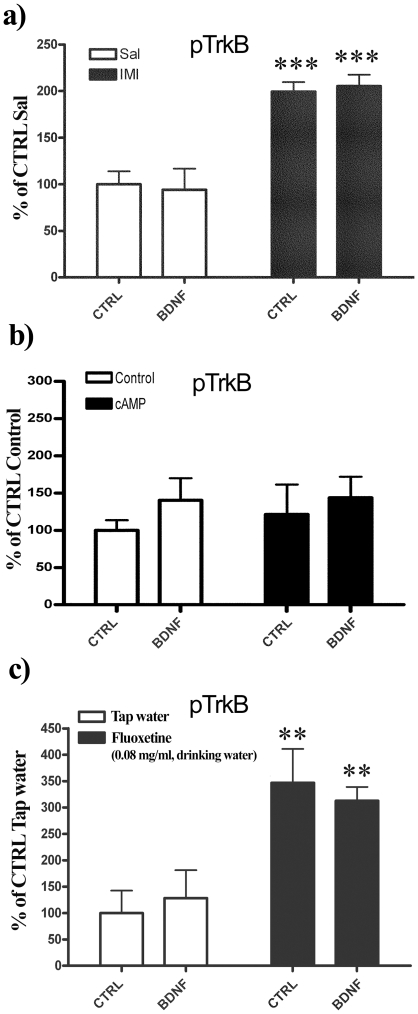
TrkB responsiveness to *ex vivo* BDNF is not altered by antidepressant drugs or cAMP signalling. (a) P60 mice were pretreated acutely with imipramine (Imi; 30 mg/kg i.p., 60 min) or saline (Sal), hippocampi were collected, sliced and incubated *ex vivo* with or without BDNF (50 ng/ml) for 5 minutes at 37°C. TrkB phosphorylation (Y816) was analyzed with western blotting. (b) P24 hippocampal microslices were pre-incubated at 37°C for 15 minutes with vehicle or sp-cAMP (10 µM) and then exposed to BDNF (50 ng/ml) or vehicle at 37°C for another 15 minutes. TrkB phosphorylation (Y705/6) was analyzed with western blotting. (c) Adult animals were chronically treated for 21 days with vehicle (Veh) or fluoxetine (0.08 mg/ml) in drinking water, hippocampi were collected, sliced and incubated *ex vivo* with or without BDNF (50 ng/ml) for 5 minutes at 37°C. TrkB phosphorylation (Y816) was analyzed with western blotting. Phospho-TrkB values are normalized against total TrkB levels. Two-Way ANOVA followed with Bonferroni *post hoc* test was performed for statistical analysis; **P<0.01, *** *P*<0.001. n = 3–6 per group.

Cyclic adenosine monophosphate (cAMP) signalling has been shown to facilitate or “gate” BDNF-induced TrkB signalling in mature hippocampal neurons *in vitro* [25]. ADs acutely increase [cAMP]i levels *via* enhancing the synaptic levels of norepinephrine (NE) and/or serotonin (5-HT) that subsequently can activate Gs –linked postsynaptic receptors. Consequently, we next tested whether acute treatment with IMI might restore the responsiveness of TrkB to BDNF *ex vivo* in adult hippocampus. As expected, IMI treatment increased the pTrkB levels in the hippocampus but no further phosphorylation was observed when BDNF was applied (**Figure 5a**). We also decided to test the potential facilitatory effect of cAMP on BDNF-TrkB signalling directly in *ex vivo* assay using cell permeable cAMP phosphodiesterase resistant cAMP analog, sp-cAMP. In microslices prepared from P24 mouse hippocampus BDNF only slightly increased the phosphorylation status of TrkB and this response was not further regulated by sp-cAMP pre-incubation (**Figure 5b**).Essentially similar findings where observed with hippocampal tissues derived from P20 mouse pups (**data not shown**).

Long-term systemic treatment with antidepressant fluoxetine has been shown to reopen developmental-like plasticity in the adult rodent brain [26], [27]. In view of this, we sought to examine the responsiveness of TrkB to BDNF in the *ex vivo* assay in brain microslices obtained from adult mice pre-treated with fluoxetine (FLX) *via* drinking water (0.08 mg/ml) for 21 days [9], [28]. In line with our previous findings [9] long-term FLX treatment increased TrkB phosphorylation in hippocampus but no further increase in pTrkB levels was detected when the microslices were incubated with BDNF (**Figure 5c**).

### BDNF activates adult brain-derived TrkB in cell-free kinase assay

**Figure 6:**
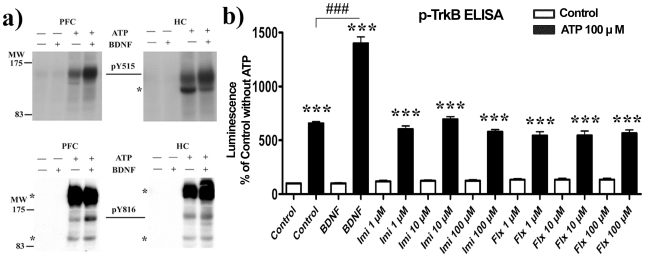
BDNF, but not antidepressant drugs, induce TrkB phosphorylation in a cell-free kinase assay. (a) Incubation of adult cortical or hippocampal brain lysates with BDNF (50 ng/ml) induces the tyrosine phosphorylation of Shc (Y515) and phospholipase-Cγ1 (Y816) binding sites of TrkB. * indicate unidentified phospho-proteins detected by the antibodies. (b) BDNF (50 ng/ml), but not imipramine or fluoxetine, induces TrkB phosphorylation in P20 mouse hippocampal lysates. Overall tyrosine phosphorylation of TrkB was analyzed using phospho-TrkB ELISA. For statistical analysis, a one-way ANOVA followed with Bonferroni *post hoc* test was performed; ***P<0.001 compared to respective control, ###*P*<0.001 compared to ATP/Control. n = 4 per group.

We used an *in vitro* cell-free kinase assay to examine whether there are any structural modifications in the TrkB receptor protein itself that might prevent BDNF from binding to and activating TrkB in the mature brain tissue. When the adult PFC and HC homogenates were subjected to the kinase assay in the presence of adenosine triphosphate (ATP, 100 µM), a clear increase in TrkB phosphorylation in response to BDNF was observed (**Figure 6a**), indicating that TrkB derived from adult brain was able to respond to BDNF under cell-free conditions. Similarly, when P20 hippocampal tissues were subjected to the kinase assay, a robust up-regulation of TrkB phosphorylation in response to BDNF was observed (**Figure 6b**). However, ADs imipramine and fluoxetine did not produce any increase in TrkB phosphorylation in the cell-free kinase assay (**Figure 6b**). Imipramine and fluoxetine also failed to alter TrkB phosphorylation when directly incubated with P20 brain microslices in the *ex vivo* assay (**Figure S3)**.

### Differential long-lasting behavioural alterations after early or late postnatal antidepressant

**Figure 7:**
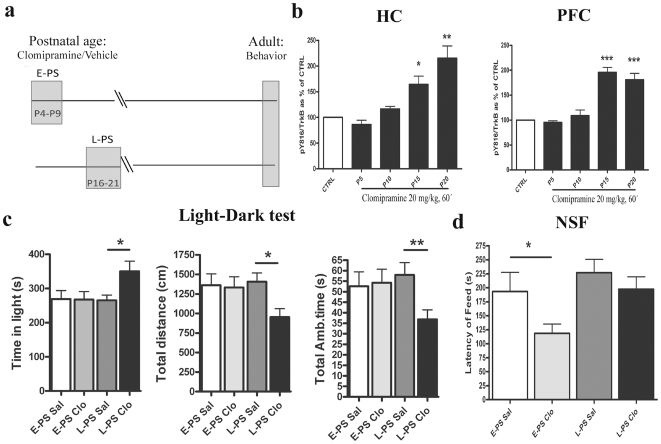
Postnatal systemic clomipramine treatments lead to long-lasting and distinct behaviours depending onexposure period. (a) Schematic figure showing treatment groups. Animals were treated with a daily dose of clomipramine (20 mg/kg, i.p.) during early (P4-9; E-PS) or late (P16-21; L-PS) postnatal period. At 3 months of age the animals were subjected to behavioural analyses (b) Age-dependent phosphorylation of TrkB receptor (Y816) in mouse brain after acute treatment with clomipramine (20 mg/g, i.p., 60 min). (c) Clomipramine treatment during late postnatal period (activates TrkB) produces anxiolytic-like behaviour in the light-dark test. (d) Clomipramine treatment during the early postnatal period (does not activate TrkB) produces anxiolytic-like behaviour in the novelty-suppressed feeding task. A t-test (b) or Two-Way ANOVA followed with Bonferroni *post hoc* test (c–d) was performed for statistical analysis; *P<0.05, ** *P*<0.01, *** *P*<0.001 compared to the respective age saline (Sal) treated group. The CTRL bar is representative of each respective control at each age. n = 6/group (biochemical analysis) or n = 10–15 per group (behavioural analysis).

Exposure of rodents to ADs during postnatal life has been shown to elicit alterations in emotional behaviour that are evident in adulthood, long after the cessation of the treatment [15]–[17], [29], [30]. We therefore tested whether the biochemical shift in TrkB responsiveness to ADs brings about differential effects on adult behaviour in response to postnatal treatment with ADs. Pups were treated with either saline or a daily dose of AD clomipramine (CLO) during two different time windows of the postnatal period: an early postnatal stage (E-PS, from P4 to P9, when TrkB is not responsive ADs) and a late postnatal stage (L-PS, from P16 to P21, when TrkB is responsive to ADs) (**Figure 7a**). CLO was selected because of its relatively short half-life after single i.p. injection and because of its prevalent serotonergic action. Furthermore, Vogel and co-workers have extensively characterized the long-term effects of early CLO treatment [15], [29]. We found that CLO activates TrkB with a similar developmental pattern to that produced by IMI (**Figure 7b**). Furthermore, CLO treatment during neither E-PS nor L-PS produced any changes in the levels of BDNF or pTrkB in the HC or PFC of adult animals (**data not shown**), suggesting that long-lasting alterations of the levels of BDNF or TrkB do not explain the behavioural phenotype observed in adult animals.

When we tested behaviour of adult animals at 3 months of age, we found that animals exposed to CLO during E-PS or L-PS, long before the behavioural testing, showed differential alterations in exploratory locomotion and anxiety-related behaviour that correlated with the age of AD treatment. Specifically, exposure to CLO treatment produced strong long-term behavioural effects in the Light-Dark test when the drug was given during the L-PS, but not when it was administered during the E-PS (**Table S1** and **Figure 7c**). Two-way ANOVA revealed a significant main effect of postnatal age x treatment interaction for the following parameters: Distance in Dark (F[1,48] = 8.94, p<0.05), Rearing in Dark (F[1,48] = 12.74, p<0.01), Total rearing (F[1,48] = 8.09, p<0.05), Ambulatory time in dark (F[1,48] = 9.76, p<0.05), Total resting time (F[1,48] = 8.34, p<0.05), Stereotypics in dark F[1,48] = 7.79, p<0.05) and Total stereotypics (F[1,48] = 8.53, p<0.05). The *post hoc* test showed that mice exposed to L-PS CLO show a significantly increased Time in the light (F[3,48] = 3,056, p<0.05, **Figure 7c**), Resting time in the light (F[3,48] = 3,025, p<0.05) and Total resting time (F[3,48] = 3,329, p<0.01). On the other hand, the same animals show reduced Time in the dark (F[3,48] = 3,209, p<0.05), Distance in light (F[3,48] = 3,001, p<0.05), Distance in dark (F[3,48] = 3,457, p<0.01), Total distance travelled (F[3,48] = 2,870, p<0.05, **Figure 7c**), Rearings in dark (F[3,48] = 4,332, p<0.01), Total rearings (F[3,48] = 2,850, p<0.05), Ambulatory time in the dark (F[3,48] = 3,195, p<0.01), Total ambulatory time (F[3,48] = 2,89, p<0.01, **Figure 7c**), numbers of stereotypics in the dark (F[3,48] = 5,082, p<0.01) and total numbers of stereotypics (F[3,48] = 4,464, p<0.01). No statistical differences were found between numbers of entries in the light, numbers of entries in the dark, total zone entries and latency to the dark (**Table S1**).

In the novelty-suppressed feeding (NSF) test, only the administration of CLO during the E-PS significantly influenced adult behaviour. In this paradigm, food-deprived mice are presented a food pellet in a novel environment and the latency to approach the food is measured [31]. Two-way ANOVA analysis showed a statistically significant effect for postnatal age (F[1,48] = 5,560, p<0.05) and treatment (F[1,48] = 4,826, p<0.05) (**Table S1** and **Figure 7d**). The *post hoc* test showed a reduced latency to feed in mice treated with CLO in the E-PS when compared to control mice at the same age (p<0.05) (**Table S1** and **Figure 7d**). A small but significant effect was shown for the home cage food consumption after the test for the postnatal age variable (F[1,48] = 5,266, p<0.05) (**Table S1**), but the *post hoc* test did not show any difference among controls and CLO treated animals. No differences were found between the groups for weight loss before the test for postnatal age and treatment (**Table S1**).

## Discussion

In the present study, we observed an age-dependent switch from a TrkB receptor readily activated by BDNF but not by ADs during early postnatal stage to a receptor activated by ADs but less sensitive to direct *ex vivo* BDNF stimulation in late postnatal stage and adulthood. AD exposure before or after this developmental switch produces differential long-lasting effects on motor and anxiety-like behaviours in adult animals. Although an inverse temporal correlation between the appearance of the responsiveness of TrkB to systemic ADs and the robust reduction of BDNF-induced TrkB phosphorylation in *ex vivo* microslices cannot be taken as any evidence of causality, it is tempting to speculate that the same maturation processes that bring about the AD responsiveness, lead to developmental changes that restrict the effects of BDNF on TrkB.

Pharmacologically, ADs such as the drugs used in the present study bind to and inhibit the monoamine reuptake pumps which lead to enhanced monoamine receptor signalling. While monoaminergic neurons are among the first neurons to be generated during early embryonic development, full maturation of these cells extends beyond postnatal life in rodents [32], [33]. Moreover, the expression and function of several monoaminergic components (receptors, reuptake pumps, and enzymes) are developmentally regulated [32], [33]. Thus, the long-lasting and differential behavioural modifications produced by age-specific early AD exposures given in this study may be explained by ongoing developmental processes in monoaminergic systems, of which some aspects could be regulated by enhanced TrkB-PLCγ1-CREB signalling during late, but not early postnatal stage. Nevertheless, these findings need to be interpreted with caution and more specific manipulations of monoaminergic systems during these specific developmental time periods are required in future studies.

AD-induced TrkB phosphorylation during late postnatal period takes place specifically at the Y816 (binding site of PLCγ1), but not at the Y515 (binding site of Shc), which is consistent to our previous findings in adult brain [8], [18]. Consequently, AD treatment induced the interaction of phosphorylated PLCγ1 with TrkB receptors and the phosphorylation of CREB, but not that of Akt [9], which is consistent of them being downstream mediators of the PLCγ1 and Shc pathways, respectively. Interestingly, CREB activation in response to IMI treatment follows the same developmental time course to that of pTrkB. CREB is considered to play a critical role in AD drug responses [34] and pCREB^Ser133^ is induced by a variety of signalling pathways, including PKA (protein kinase A), Ca^2+^-calmodulin kinase II and Mitogen-activated protein kinase pathways [35]. Activation of the PKA pathway through G-protein coupled monoamine receptors is considered a central mechanism for CREB phosphorylation in response to ADs [34]. The tight coupling of the developmental time course of the responsiveness of TrkB and CREB to ADs suggest that the activation of TrkB is a prerequisite for the CREB phosphorylation by ADs.

We have here confirmed and extended the unexpected observations previously reported by Knüsel *et al* [13] demonstrating that incubation of brain microslices prepared from adult rodent brain in the presence of BDNF fails to induce a pTrkB response, while a robust autophosphorylation is observed in microslices prepared from embryonic or early postnatal brain. Under identical conditions, a closely related neurotrophin NGF readily induced phosphorylation of its receptor TrkA in brain microslices prepared from mature brain tissues, suggesting that the dramatic reduction in the responsiveness of TrkB to BDNF in a mature tissue is not related to a reduced penetrance of the neurotrophin or to any general unsuitability of the assay for adult brain tissues. Furthermore, basal TrkB phosphorylation status in the hippocampus of BDNF deficient mice was significantly reduced during early postnatal stage whereas adult BDNF deficient mice showed no changes in pTrkB levels when compared to control mice, as also reported before [21].

It was previously suggested that the developmentally regulated increase in the truncated TrkB.T1 receptor that can act as dominant-negative partner for full-length TrkB, might explain the specific reduction of the responsiveness of TrkB to BDNF. We ruled out this possibility, since hippocampal TrkB receptors responded similarly to BDNF and ADs in the tissue samples prepared from WT and *trkB.T1* ^−/−^ mice. Another possible explanation could be that the ability of BDNF to induce TrkB activation in the mature brain depends on additional gating mechanisms such as cAMP or adenosine [25], [36]. To test this possibility, we investigated whether systemic AD treatment, which is known to activate [cAMP]_i_ signalling, might facilitate or restore the ability of BDNF to activate TrkB, but this did not turn out to be the case. Moreover, direct up-regulation of [cAMP]_i_ signalling in the *ex vivo* assay failed to facilitate BDNF-induced TrkB phosphorylation in mature microslices. It is possible that functional maturation of neuronal networks and closure of sensitive periods may modify the responsiveness of TrkB to BDNF (and ADs). Chronic treatment with AD fluoxetine has been shown to reopen developmental-like plasticity in the adult rodent cortex [26], but also this treatment failed to facilitate the responsiveness of TrkB to *ex vivo* BDNF in the adult mouse brain. However, the cell-free TrkB kinase assay suggests that there does not appear to be any structural modifications in the TrkB protein itself that would prevent the receptor from binding to and being activated by BDNF.

The reason for and significance of the apparently complete loss of TrkB responsiveness to BDNF (also to high concentrations, see [13]) in an *ex vivo* assay after early postnatal life remains unclear. Although recent evidence suggest that lack of BDNF has only minor effects on the survival and structure of cortical neurons in the CNS [37], BDNF has, nevertheless, well characterized actions in the adult CNS. For example, loss of BDNF produces clear behavioural effects in adulthood [20], [38], [39]. In addition, *in vivo* infusion of BDNF into the adult brain increases TrkB phosphorylation, albeit only at relatively high concentrations [40], and produces distinct behavioural responses depending on the site of injection [10], [11], [41] and at least part of these effects are mediated *via* TrkB [11]. Furthermore, we have observed an essentially identical loss of behavioural responses to ADs in both *bdnf* ^+/−^ mice and in mice over-expressing an inhibitory form of *trkB* (TrkB.T1) in brain [8], clearly implicating the BDNF-TrkB signalling in this response. Thus, even though the mechanisms responsible for this discrepancy remain unclear and require further studies, these results clearly demonstrate that a change in TrkB responsiveness takes place during brain maturation at around 2 weeks of age in mice.

Previous studies have demonstrated that exposure of rodents to different ADs during early postnatal development brings about long-lasting biochemical and behavioural effects which can be observed in adult animals, long after the drug has disappeared from the body [15]–[17], [29], [30]. In view of the differential developmental responsiveness of TrkB to ADs during postnatal age we sought to examine potential long-lasting behavioural alterations produced by sub-chronic CLO treatment during the period when the drug does or does not activate TrkB (P16-21 or P4-9, respectively). Interestingly, we found that mice treated with CLO during the P4-9 show long-lasting behavioural changes in the novelty suppressed feeding test that were not observed in animals treated during the P16-21. Conversely, the light dark test revealed long-lasting behavioural alterations that were observed only in mice treated with CLO during P16-21.

In conclusion, our present data suggest the intriguing hypothesis that in rodents, TrkB shows contrasting responsiveness to BDNF and ADs during the postnatal maturation and that this regulation may have important repercussions on the development of an adult behavioural phenotype.

## Materials and Methods

### Animals

Male C57BL/6JRccHsd mice (Harlan Laboratories, Netherlands), *trkB.T1* ^−/−^ [24], *Bdnf* ^+/−^ */Bdnf* ^−/−^ [19] or BDNF^2L/2LCk-cre^ [20] and their wild-type littermates were used for the studies. The animals were kept under standard laboratory conditions (21°C, 12 h light-dark cycle, lights on at 6 A.M.). All the experiments were carried out according to the guidelines of the Society for Neuroscience and were specifically approved by the County Administrative Board of Southern Finland (Permit: ESLH-2007- 09085/Ym-23).

### Experimental Design

#### Postnatal acute antidepressant treatments

Dams with their litters were housed individually. For postnatal acute antidepressant treatments, age-matched litters (P5-21) were randomly assigned to receive i.p. injection of saline (SAL) (NaCl 0.9%, 5 ml/kg), imipramine (IMI) (HCl salt, dissolved in SAL, 30 mg/kg, 5 ml/kg; Sigma-Aldrich Finland Oy, Helsinki), or clomipramine (HCl salt, dissolved in SAL, 20 mg/kg, 5 ml/kg; Sigma-Aldrich) and following indicated lag-time (30–60 min) hippocampus and medial prefrontal cortex were collected as described in [9]. Briefly, mice were stunned with CO_2_, the brains quickly removed and bilateral hippocampus and prefrontal cortex were dissected out on a dish cooled on dry-ice. Samples were homogenized in a NP++ buffer (300 µl/sample; composition: 137 mM NaCl, 20 mM Tris, 1% NP-40, 10% glycerol, 48 mM NaF, H_2_O, 2× Complete inhibitor mix (Roche) and 2 mM Na_3_VO_4_). After incubation on ice for 15 min, samples were centrifuged (16100 *g*, 15 min, +4°C) and the supernatant collected for further analysis.

#### Postnatal sub-chronic clomipramine treatment

Dams with their litters were housed individually. The litters were randomly assigned to 4 groups: saline injected (SAL) (NaCl 0.9%, 5 ml/kg), and clomipramine injected (CLO) (dissolved in SAL, 20 mg/kg, 5 ml/kg) [16], starting at postnatal day 4 (P4) until P9 (Early-Postnatal Stage, E-PS), and from P16 to P21 (Late-Postnatal Stage, L-PS). Each pup was weighted and injected once daily (between 9 A.M.–11 A.M.). During the treatment the litter was removed from its home cage and placed in a bucket with some shavings of their home cage. All the pups belonging to a single litter were injected randomly in less than 3 minutes. After the injection the pups were immediately placed back in their home cage. CLO treatments did not have any significant effect on the weight gain of the pups and later in adulthood (**Figure S4**).

Mice were weaned on P22 and males were housed together (4–6 mice/cage) until the behavioural experiments. For biochemical analyses the animals were killed after 2 weeks of the last behavioural test and their tissues were collected and processed as described before.

#### Adult antidepressant treatment

For the acute AD treatments, adult (∼P90) mice received a single i.p. injection of imipramine (dissolved in SAL, 30 mg/kg, 5 ml/kg) or SAL and were killed 30 minutes after. For chronic AD treatment, adult (∼P90) mice had free access to either tap water or to 0.08 mg/ml solution of fluoxetine (FLX; HCl salt; Orion Pharma, Turku, Finland) for 21 days [9], [28]. On the final day of treatment the animals were killed. Hippocampus and prefrontal cortex samples were rapidly dissected out and processed as described before.

### Ex vivo assays

The *ex vivo* BDNF stimulation assay was performed according to Knüsel *et al* [13] with slight modifications. Mouse hippocampi and medial prefrontal cortex at different ages were dissected. After dissection the samples were placed on a filter paper wet with cold Neurobasal Medium (NBM) (Neurobasal medium (Gibco), 2% B27 supplement (Gibco), 0.5 mM Glutamine (Gibco) and penicillin/streptomycin (Sigma), and then the tissues were sliced into equally sized pieces. The slices were transferred in fresh tubes and washed twice with NBM +10% heat-inactivated Fetal Calf Serum (FCS) (Gibco). Tissues were gently re-suspended with a Pasteur pipette. The medium was removed and 600 µl of NBM +10% FCS with or without BDNF (Peprotech) or NGF (Promega) were added. The tubes were closed and incubated at +37°C for 5 or 15 minutes gently shaking. Finally the tubes were put on ice, spun down, the medium removed, the pellet was rinsed once with PBS and then the samples were homogenized in NP++ buffer. A set of samples were pre-incubated with cAMP analog sp-cAMP (10 µM; Sigma-Aldrich) for 15 minutes at +37°C before BDNF stimulation.

### TrkB kinase activity assay

Assays were performed according to the procedures described by Angeles *et al* [42] with some modifications. Each assay was performed in a final volume of 20 µL of 50 mM Hepes pH 7.4, 140 mM NaCl, 10 mM MnCl_2_, 0.05% BSA, 2% DMSO with or without 100 µM ATP and/or 50 ng/mL BDNF. The reaction was initiated adding 40 µg of NP++ lyzed protein extract to the mix and the incubation was allowed to proceed for 10 min at 37°C. For a subset of samples, the reaction was quenched by adding an equal volume of 4× Laemmli sample buffer and proteins were separated by SDS-PAGE and TrkB phosphorylation status analyzed with western blotting as described below. Rest of the samples we immediately transferred to Trk antibody containing ELISA (enzyme-linked immunosorbent assay) plates, 3% BSA/PBS-T (+2 mM Na_3_VO_4_) added *ad* 200 µl, and phospho-TrkB ELISA proceeded as previously described [18].

### Immunoprecipitation and western blot

Sample protein concentrations were measured using the Lowry Method (Bio-Rad DC protein assay). Lectin precipitation was carried out essentially as described [9] using Wheat Germ Agglutinin (EY Laboratories, San Matteo, CA, USA). TrkB immunoprecipitation was carried out using a TrkB specific antibody (5 µl/sample; #AF1494, R&D Systems Europe, Abingdon, UK) in conditions described in [9]. Proteins were separated in a SDS-PAGE under reducing conditions and blotted to a PDVF membrane (300 mA for 1 h at 4°C). Membranes were incubated with the following primary antibodies: anti-p-TrkB^Y816^ (1∶5000; kind gift from Dr. M. Chao, Skirball Institute, NY, USA), anti-p-TrkA/B^Y490/Y515^ (#9141; 1∶2000; Cell Signalling Technology (CST), MA, USA), anti-p-TrkA/B^Y674-5/Y705-6^ (1∶1000; CST), anti-TrkB_out_ (#610102; 1∶2000; RD Transduction Laboratories, Franklin Lakes, NJ USA), anti-p-CREB^S133^ (#9198; 1∶1000; CST), anti-CREB (sc-186; 1∶100, Santa Cruz Biotechnology (SCB), CA, USA), anti-p-Akt^Thr308^ (#9275; 1∶2000, CST), anti-AKT (#4691; 1∶1000, CST), anti-p-PLCγ1^Y783^ (#2821, 1∶1000, CST) or anti-Trk (#sc-11 (rabbit), 1∶1000, SCB). Further the membranes were washed with TBS/0.01% Tween® (TBST) and incubated with horseradish peroxidase conjugated secondary antibodies (1∶10000 in non fat dry milk, 1 h, RT, Bio-Rad Laboratories, Hercules, CA). After subsequent TBST washes, secondary antibodies were visualized using electrochemiluminescence kits (Amersham Biosciences) followed by an exposure to Fuji LAS-3000 Camera (Tamro Medlabs, Vantaa, Finland) for ECL detection.

### Behavioural tests

Battery of behavioural tests [43] were performed on adult male E-PS, L-PS and control mice, starting from P90. The tests were done between 9 A.M.-3 P.M. with at least 3 days of interval between each test. Exploratory locomotion, depression- and anxiety-like behaviours were assessed by the following tests: light-dark test, elevated plus maze, open-field test, forced swim test and novelty-suppressed feeding test. The tests were performed in this order in a period of 3 weeks [43]. Among all these tests, only the light-dark test and the novelty-suppressed feeding test showed statistically significant differences between the drug and saline treated groups, and only these two tests are described in more detail in this manuscript.

#### Light-Dark Test (LD)

Testing was performed for 10 min in an acrylic cage (28.5×28.5×20 cm) (TSE, Bad Homburg, Germany) divided into two equal sized compartments: one part with transparent walls, open topped and brightly illuminated (∼450 lx by a 40 W light bulb fixed 55 cm above the floor), the other part made from black plastic (passing infrared light) and covered by a lid. The two compartments were separated by a partition containing an opening (7×5 cm) in its centre at floor level. The animal was placed in the centre of the light compartment facing away from the opening, and the latency to enter dark area, time spent in the compartments, total distance travelled, immobility time and the number of entries to dark were measured over 10 min. In addition, rearing time was also calculated. Testing apparatus was thoroughly cleaned before each animal using 70% ethanol.

#### Novelty-Suppressed Fedding

Novelty-Suppressed Feeding: The Novelty-Suppressed Feeding (NSF) was performed as previously described [30]. Briefly, the test was performed in brightly lit (800–900 lux) open arena (51×35 cm). A small piece of filter paper with a pre-weighted food pellet was placed in the middle of the arena. The animals were deprived of food for 24 h with water available *ad libitum*. On testing day each animal was removed from its home cage and placed in a holding cage for 30 min before the test and then put in one corner of the arena. The latency to beginning of a feeding episode was measured (maximum over a period of 5 min). Immediately, after starting of feeding the mouse was removed from the arena and placed with the food pellet in its home cage and allowed to feed *ad libitum* over a period of 5 min. The amount of food consumed was quantified by weighting the pellet.

### Data analysis

Immunoblot bands were quantified using NIH ImageJ. All the data are expressed as mean ± SEM (Standard Error of Mean) and as percentage of control. Statistical analyses were performed using GraphPad Prism 4.0 for Windows (GraphPad Software, San Diego California USA). For comparison between two groups Two-way Student's t-test was used. Two-way ANOVA or two-way ANOVA for repeated measures was used to reveal main effect and interaction between the factors followed by Bonferroni *post hoc* test. The criterion for significance was set to p<0.05.

## Supporting Information

Figure S1 **Effect of imipramine on the phosphorylations of TrkB Shc binding site and Akt during development.** (a) Phosphorylation of TrkB Shc binding site (Y515) after acute imipramine treatment (30 mg/kg, 30 min, i.p.) in prefrontal cortex (PFC) and hippocampus (HC). (b) Phosphorylation of Akt (Thr^308^) after acute imipramine treatment (30 mg/kg, 30 min, i.p.) in prefrontal cortex (PFC) and hippocampus (HC). Phospho-protein values are normalized against corresponding total protein levels. Results are expressed as percentage of respective control. A t-test was performed between each control and treated group of animals at the different ages. n = 6–7 per group.
(TIF)

Figure S2 **Acute BDNF stimulations does not regulate total TrkB protein levels in brain microslices.** Representative blots showing the levels of full-length TrkB after control or BDNF stimulation in cortical (PFC) or hippocampal (HC) microslices prepare from P5-P60 old mice.
(TIF)

Figure S3 **Imipramine and fluoxetine does not directly induce TrkB phosphorylation in hippocampal microslices** ***ex vivo*** **.** P20 hippocampal microslices were incubate with vehicle or different concentrations of imipramine (Imi; 1–100 µM) (a) or fluoxetine (Flx; 1–100 µM) (b) for 15 min at 37°C and TrkB phosphorylation (Y705/6) analyzed with western blotting. n = 3 per group.
(TIF)

Figure S4 **The effect of postnatal clomipramine on weight gain of mouse.** A daily dose of clomipramine (20 mg/kg, i.p.) or saline during early (P4-9; E-PS) or late (P16-21; L-PS) postnatal period produced no changes on weight gain during (P4-21) (a) or after the treatments (P28-120) (b). n = 10–15 per group.
(TIF)

Table S1 **Postnatal treatments with clomipramine lead to long-lasting and distinct behaviours depending on early exposure period.** The effect of early (postnatal days 4–9) and late (postnatal days 16–21) postnatal clomipramine treatment (20 mg/kg, i.p., once daily) on behaviour in light-dark box test and novelty suppressed feeding test in adult (P90) animals. Two-Way ANOVA followed with Bonferroni *post hoc* test was performed for statistical analysis; *P<0.05, ** *P*<0.01. n = 10–15 per group.
(DOCX)
