# Supplementary figures and images for: The Responsiveness of TrkB to BDNF and Antidepressant Drugs Is Differentially Regulated during Mouse Development
Source: PLoS One. 2012 Mar 2;7(3):e32869. doi: 10.1371/journal.pone.0032869 (PMC3292581; doi:10.1371/journal.pone.0032869)

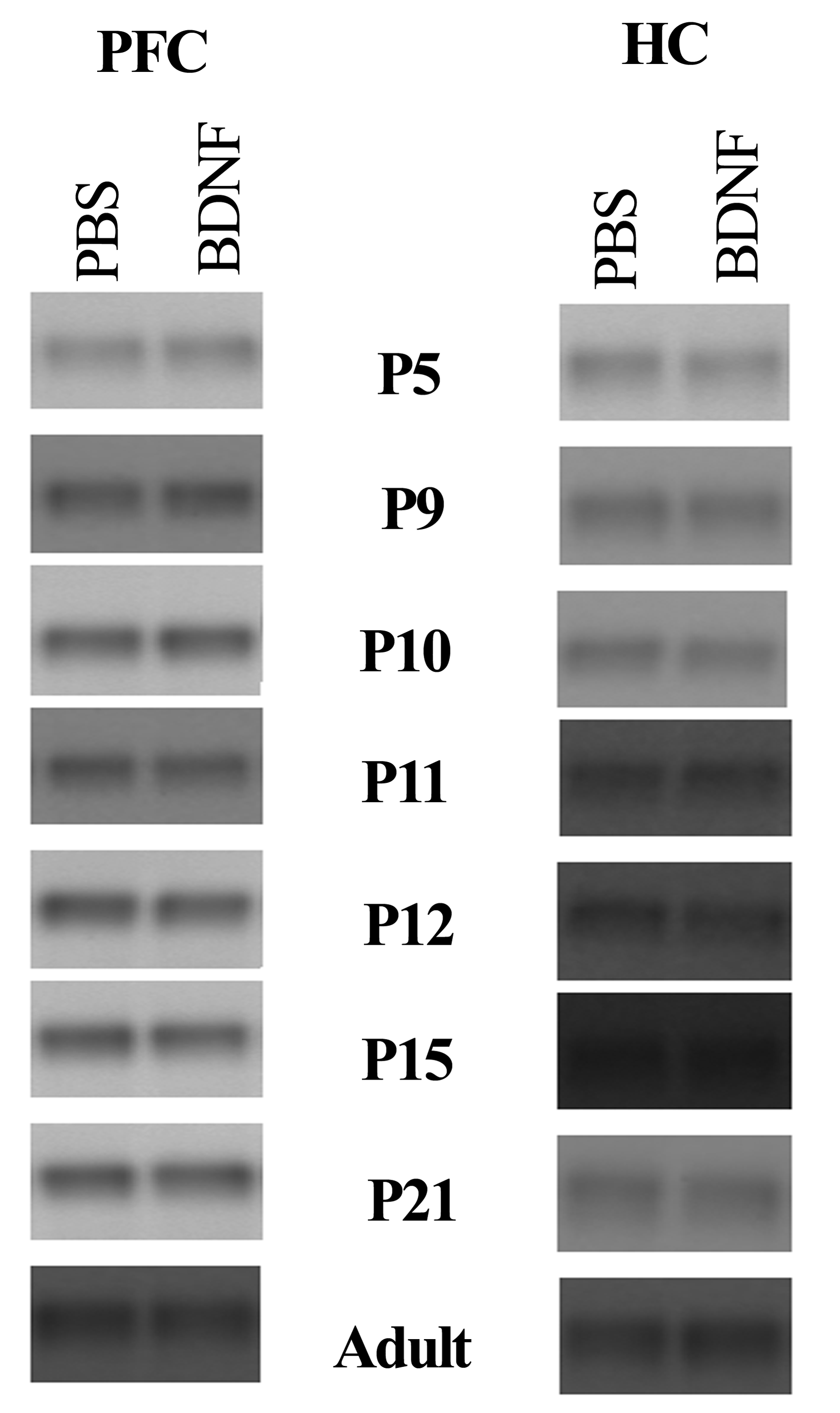

Supplement: Figure S2 — Acute BDNF stimulations does not regulate total TrkB protein levels in brain microslices. Representative blots showing the levels of full-length TrkB after control or BDNF stimulation in cortical (PFC) or hippocampal (HC) microslices prepare from P5-P60 old mice. (TIF) [file pone.0032869.s002.tif]

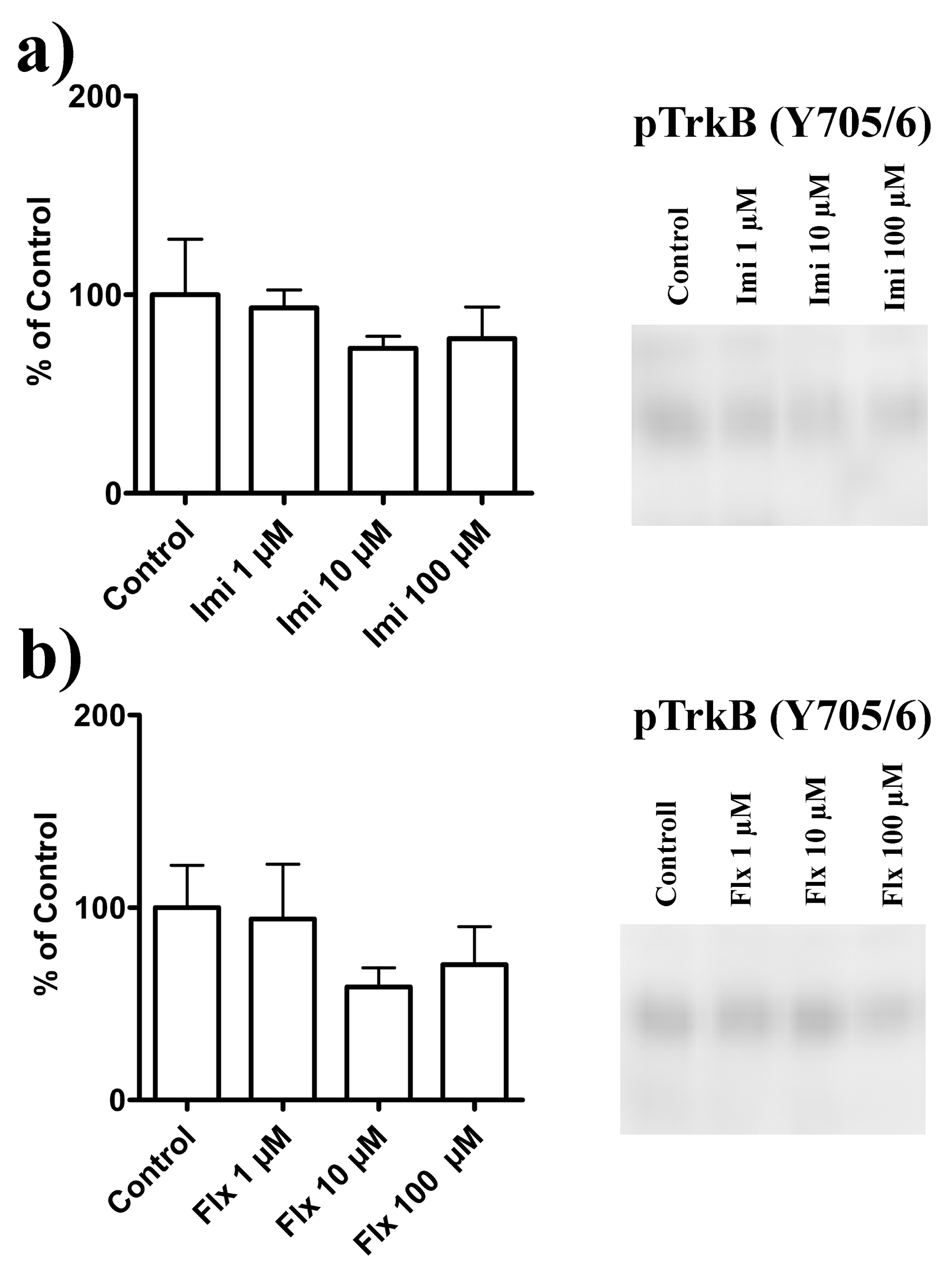

Supplement: Figure S3 — Imipramine and fluoxetine does not directly induce TrkB phosphorylation in hippocampal microslices ex vivo . P20 hippocampal microslices were incubate with vehicle or different concentrations of imipramine (Imi; 1–100 µM) (a) or fluoxetine (Flx; 1–100 µM) (b) for 15 min at 37°C and TrkB phosphorylation (Y705/6) analyzed with western blotting. n = 3 per group. (TIF) [file pone.0032869.s003.tif]

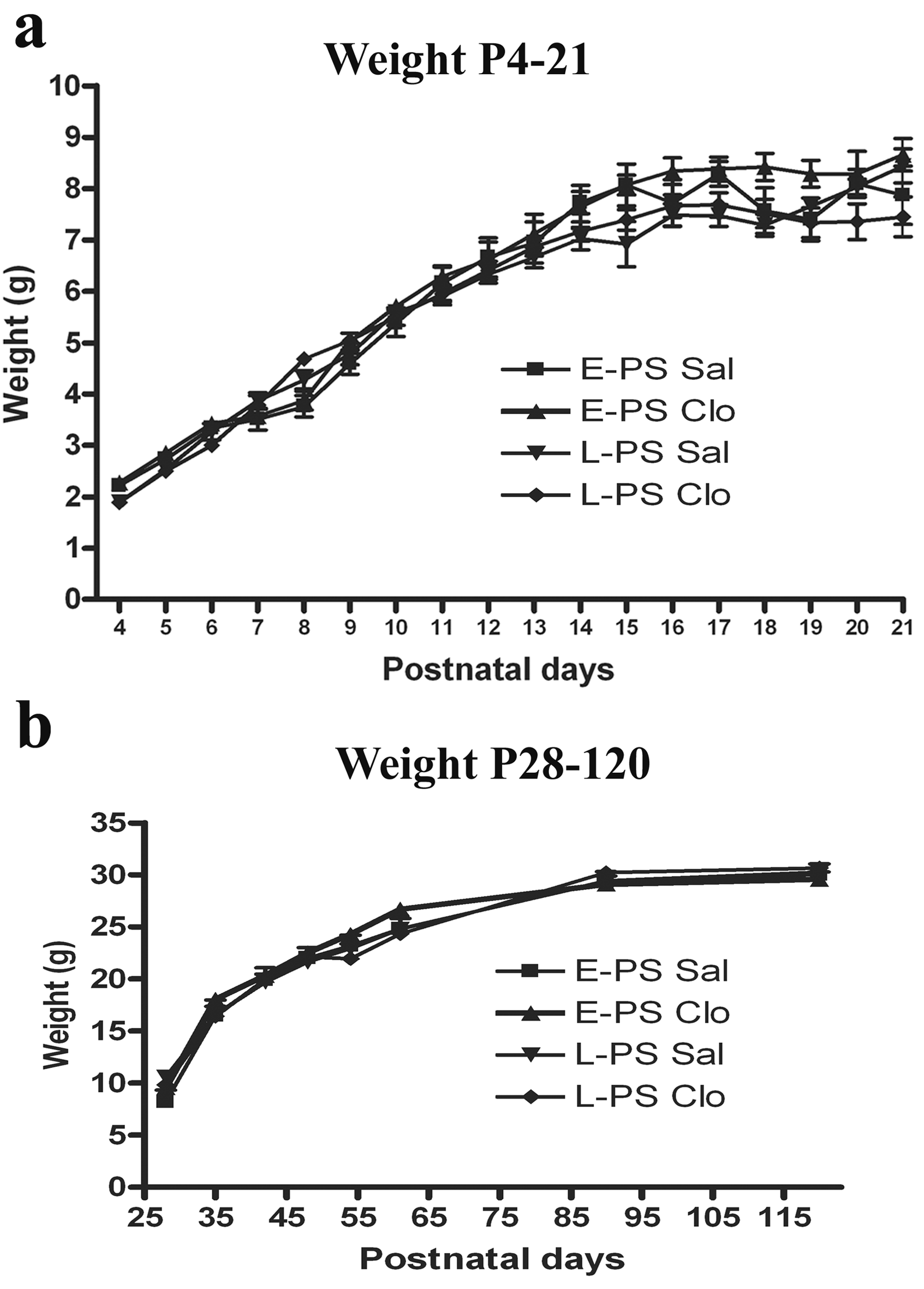

Supplement: Figure S4 — The effect of postnatal clomipramine on weight gain of mouse. A daily dose of clomipramine (20 mg/kg, i.p.) or saline during early (P4-9; E-PS) or late (P16-21; L-PS) postnatal period produced no changes on weight gain during (P4-21) (a) or after the treatments (P28-120) (b). n = 10–15 per group. (TIF) [file pone.0032869.s004.tif]
