# Supplementary material for: The Responsiveness of TrkB to BDNF and Antidepressant Drugs Is Differentially Regulated during Mouse Development
Source: PLoS One. 2012 Mar 2;7(3):e32869. doi: 10.1371/journal.pone.0032869 (PMC3292581; doi:10.1371/journal.pone.0032869)
Supplement: Table S1 — Postnatal treatments with clomipramine lead to long-lasting and distinct behaviours depending on early exposure period. The effect of early (postnatal days 4–9) and late (postnatal days 16–21) postnatal clomipramine treatment (20 mg/kg, i.p., once daily) on behaviour in light-dark box test and novelty suppressed feeding test in adult (P90) animals. Two-Way ANOVA followed with Bonferroni post hoc test was performed for statistical analysis; *P<0.05, ** P<0.01. n = 10–15 per group. (DOCX) [file pone.0032869.s005.docx]

|  | |  |  |  |  |  |  |  |
| --- | --- | --- | --- | --- | --- | --- | --- | --- |
| Test and Parameter | | **E-PS**  **CTRL**  **(n=10)** | **E-PS**  **CLO**  **(n=15)** | **L-PS**  **CTRL**  **(n=12)** | **L-PS**  **CLO**  **(n=15)** | Postnatal Stage | Treatment | Interaction |
| **LIGHT-DARK BOX** | |  |  |  |  |  |  |  |
| Time in Light (s) | | 269,0 ± 24,9 | 267,5 ± 22,9 | 265,4 ± 15,1 | 350,2 ± 29,7***** | ns | ns | ns |
| Time in Dark (s) | | 334,8 ± 27,1 | 336,7 ± 17,1 | 321,7 ± 14,8 | 250,0 ± 31,1***** | ns | 0,0980 | ns |
| Latency to dark (s) | | 83,1 ± 23,8 | 117,4 ± 24,9 | 119,3 ± 21,1 | 166,4 ± 31,3 | ns | ns | ns |
| Distance in Light (cm) | | 610,0 ± 77,0 | 532,5 ± 56,7 | 581,4 ± 46,2 | 397,5 ± 39,5***** | ns | ns | ns |
| Distance in dark (cm) | | 730,2 ± 69,1 | 759,6 ± 79,9 | 825,2 ± 72,0 | 519,5 ± 63,8****** | ns | 0,0681 | 0,0282 |
| Total Distance (cm) | | 1298 ± 145,0 | 1332 ± 137,8 | 1407 ± 112,2 | 952,2 ± 108,7***** | ns | ns | 0,0642 |
| Entries to Light | | 30,7 ± 3,5 | 32,3 ± 3,6 | 30,2 ± 2,4 | 30,5 ± 4,4 | ns | ns | ns |
| Entries to Dark | | 36,1 ± 3,4 | 34,2 ± 3,1 | 29,8 ± 3,6 | 32,2 ± 3,9 | ns | ns | ns |
| Total Zone Entries | | 66,8 ± 6,2 | 66,5 ± 6,6 | 60,0 ± 5,7 | 62,7 ± 8,0 | ns | ns | ns |
| Rearing in Light | | 34,9 ± 9,6 | 42,4 ± 8,0 | 36,4 ± 3,9 | 23,3 ± 4,6 | ns | ns | ns |
| Rearing in Dark | | 29,3 ± 4,6 | 35,2 ± 4,1 | 34,0 ± 3,4 | 18,2 ± 3,2****** | ns | ns | 0,0081 |
| Total Rearing | | 64,2 ± 13,6 | 77,6 ± 10,9 | 70,5 ± 6,7 | 41,6 ± 7,6***** | ns | ns | 0,0398 |
| Resting Time in Dark (s) | | 208,8 ± 24,2 | 190,9 ± 16,3 | 189,5 ± 11,4 | 161,6 ± 23,0 | ns | ns | ns |
| Ambulatory Time in Light (s) | | 23,7 ± 3,6 | 22,6 ± 2,8 | 24,0 ± 2,5 | 16,5 ± 2,2 | ns | ns | ns |
| Ambulatory Time in Dark (s) | | 28,8 ± 3,4 | 31,5 ± 3,8 | 33,8 ± 3,5 | 20,3 ± 2,5****** | ns | ns | 0,0230 |
| Total Ambulatory Time (s) | | 52,5 ± 6,8 | 54,2 ± 6,4 | 57,9 ± 5,9 | 36,8 ± 4,4****** | ns | ns | 0,0657 |
| Resting Time in Light (s) | | 162,5 ± 20,6 | 172,5 ± 21,0 | 170,4 ± 14,8 | 248,8 ± 31,2***** | 0,0860 | ns | ns |
| Total Resting Time (s) | | 371,3 ± 12,3 | 363,5 ± 13,8 | 360,0 ± 11,1 | 410,4 ± 13,7****** | ns | ns | 0,0343 |
| Stereotypics in Light | | 837,6 ± 83,8 | 880,1 ± 63,6 | 858,6 ± 36,8 | 843,9 ± 60,6 | ns | ns | ns |
| Stereotypics in Dark | | 1259 ± 60,5 | 1250 ± 84,1 | 1278 ± 62,1 | 902,2 ± 97,7****** | 0,0540 | 0,0255 | 0,0327 |
| Total Stereotypics | | 2097 ± 70,2 | 2131 ± 88,7 | 2137 ± 67,1 | 1746 ± 115,3****** | 0,0715 | 0,0623 | 0,0279 |
|  |  | |  |  |  |  |  |  |
|  |  | |  |  |  |  |  |  |
| Test and Parameter | **E-PS**  **CTRL**  **(n=10)** | | **E-PS**  **CLO**  **(n=15)** | **L-PS**  **CTRL**  **(n=12)** | **L-PS**  **CLO**  **(n=15)** | Postnatal Stage | Treatment | Interaction |
| **NOVELTY-SUPPRESSED FEEDING** |  | |  |  |  |  |  |  |
| Latency of Feed (s) | 193,3 ± 34,3 | | 118,5 ± 16,7***** | 226,9 ± 23,8 | 197,1 ± 22,3 | 0,0225 | 0,0329 | ns |
| Homecage food consumption (g) | 0,14 ± 0,016 | | 0,15 ± 0,01 | 0,10 ± 0,02 | 0,11 ± 0,01 | 0,0262 | ns | ns |
| Weight Loss (%) | 7,20 ± 0,2 | | 7,41 ± 0,4 | 8,13 ± 0,6 | 6,68 ± 0,3 | ns | ns | 0,0522 |
